# Early Genital Surgery in Disorders/Differences of Sex Development: Patients’ Perspectives

**DOI:** 10.1007/s10508-021-01953-6

**Published:** 2021-03-12

**Authors:** Elena Bennecke, Stephanie Bernstein, Peter Lee, Tim C. van de Grift, Agneta Nordenskjöld, Marion Rapp, Margaret Simmonds, Jürg C. Streuli, Ute Thyen, Claudia Wiesemann, the dsd-LIFE Group

**Affiliations:** 1 Department of Paediatric Endocrinology and Diabetology, Charité-Universitätsmedizin Berlin, Corporate Member of Freie Universität Berlin, Humboldt-Universität zu Berlin, and Berlin Institute of Health, Berlin, Germany; 2 Sozialpädiatrisches Zentrum, Center for Chronically Sick Children, Charité-Universitätsmedizin Berlin, Corporate Member of Freie Universität Berlin, Humboldt-Universität zu Berlin, and Berlin Institute of Health, Berlin, Germany; 3 Department of Paediatrics, Göttingen University Medical Center, Göttingen, Germany; 4 Department of Pediatrics, Penn State College of Medicine, Hershey, PA USA; 5 Department of Plastic, Reconstructive and Hand Surgery, Amsterdam University Medical Center (VUmc), Amsterdam, The Netherlands; 6 Department of Medical Psychology and Sexology, Amsterdam University Medical Center, Amsterdam (VUmc), The Netherlands; 7 Pediatric Surgery, Karolinska University Hospital and Department of Women’s and Children’s Health, and Center of Molecular Medicine, Karolinska Institutet, Stockholm, Sweden; 8 Klinik für Kinder- und Jugendmedizin, Universität zu Lubeck, Lubeck, Germany; 9 Androgen Insensitivity Syndrome Support Group, London, UK; 10 Institute of Biomedical Ethics and History of Medicine, University of Zurich, Zürich, Switzerland; 11 Department of Medical Ethics and History of Medicine, Göttingen University Medical Center, Humboldtallee 36, 37073 Göttingen, Germany

**Keywords:** Disorders of sex development, Congenital adrenal hyperplasia, Genital surgery, Pediatrics, Ethics

## Abstract

Controversy continues over a proposed moratorium on elective genital surgery in childhood for disorders/differences of sex development (DSD). Empirical evidence on patient preference is needed to inform decision-making. We conducted a multicentre survey by cross-sectional questionnaire in 14 specialized clinics in six European countries. The sample comprised 459 individuals (≥ 16 years) with a DSD diagnosis, including individuals with congenital adrenal hyperplasia (CAH) (*n* = 192), XY DSD with prenatal androgen effect (A) (*n* = 150), and without (nA) (*n* = 117). Main outcome measures were level of agreement with given statements regarding genital surgery, including clitoris reduction, vaginoplasty, and hypospadias repair. A total of 66% of individuals with CAH and 60% of those with XY DSD-A thought that infancy or childhood were the appropriate age for genital surgery. Females with XY DSD were divided on this issue and tended to prefer vaginoplasty at a later age (XY DSD-A 39%, XY DSD-nA 32%). A total of 47% of males preferred early hypospadias surgery. Only 12% (CAH), 11% (XY DSD-A), and 21% (XY DSD-nA) thought they would have been better off without any surgery in childhood or adolescence. Individuals who had early genital surgery were more likely to approve of it. Outcome data failed to support a general moratorium on early elective genital surgery. Participant perspectives varied considerably by diagnostic category, gender, history of surgery, and contact with support groups. Case-by-case decision-making is better suited to grasping the ethical complexity of the issues at stake.

*Trial registration*: German Clinical Trials Register DRKS00006072.

## Introduction

Controversy continues over elective surgery in childhood for disorders/differences of sex development (DSD) (Carmack, Notini, & Earp, 2016; Hughes, Houk, Ahmed, Lee, & LWPES/ESPE Consensus Group, 2006; Lee et al., 2016; Mouriquand et al., 2016). Critics demand a general moratorium on surgery designed to “normalize” ambiguous genitalia, as in congenital adrenal hyperplasia (CAH), partial androgen insensitivity syndrome (pAIS), and gonadal dysgenesis (GD), buttressing their arguments by patients’ experience of pain, humiliations, and poor functional outcome, as well as the perceived violation of human rights (Diamond & Garland, 2014; Organisation Intersex International Australia et al., 2017). Surgery that is “cosmetic rather than vital for health” and, thus, considered “medically unnecessary” should be postponed until the age of informed consent. Major political institutions, such as the European Parliament, the Council of Europe, Commissioner for Human Rights, and the Special Rapporteur of the United Nations on torture and other cruel, inhuman or degrading treatment or punishment, have recently adopted this position (Council of Europe, Commissioner for Human Rights, 2015; European Parliament, 2013; Human Rights Council, 2013). German and Swiss ethical boards, along with other interdisciplinary ethical committees, have taken a more cautious stand, allowing for case-by-case decision-making in early childhood (German Ethics Council, 2012; Swiss National Advisory Committee, 2012). However, given the political weight of the moratorium advocates, an outright ban on “normalizing” early surgery may be implemented into national laws sooner or later. The question arises whether substantial evidence on patients’ perspectives is available to support such a significant change in medical practice.

Medical decision-making involves value judgements on well-being, identity, and normality, not only by healthcare professionals but also by patients (Bennecke et al. 2020; Brinkmann, Schuetzmann, & Richter-Appelt, 2007; Köhler et al., 2012). It is unclear whether DSD patients are as dissatisfied with early genital surgery as critics maintain (Meyer-Bahlburg et al., 2004; Zucker, 2002). Given the potential impact of a moratorium, we felt an urgent need for more substantial evidence of patients’ views for and against such surgery (Sandberg, Callens, & Wisniewski, 2015). The objective of this study was to assess the impact of gender, DSD condition, experience of early surgery, and support group contact on these perspectives using data from a quantitative cross-sectional patient-reported outcome (PRO) study by the dsd-LIFE Network in six European countries.

## Method

### Participants

Dsd-LIFE is a multicentre cross-sectional clinical evaluation study mobilizing 16 partners in Germany, France, the Netherlands, Poland, Sweden, and the United Kingdom, of whom 14 were active recruiting sites. The methodological approach, including the process to ensure valid translation, is described in Rohle et al. (2017). Adolescents (≥ 16 years) and adults with DSD were recruited from February 2014 to September 2015 via medical centers, the project website, and support group contact. All had a clinically confirmed DSD diagnosis conforming to the Chicago Consensus Conference Guidelines (Hughes et al., 2006). The first part of the dsd-LIFE study included a medical interview, a retrospective chart review, and medical examination. The second part of the study was a patient-reported outcome questionnaire. Informed consent was obtained from all individual participants included in the study.

### Procedure

In this substudy, we evaluated 6 items related to views on elective surgery of participants with 46,XX karyotype and CAH, 45,X/46,XY and 46,XY DSD as they often undergo genital surgery in childhood and/or adolescence. We classified them into three subgroups according to diagnosis and exposure to prenatal androgen effect: those with CAH and 46,XX karyotype (CAH), those with 46,XY or 45,X/46,XY karyotype and prenatal androgen effect (XY DSD-A), and those with 46,XY karyotype or 45,X/46,XY without prenatal androgen effect (XY DSD-nA) (Table 1). In the XY DSD group, we chose exposure to prenatal androgen effect as a diagnostic criterion as it induces atypical development of the external and/or internal genitalia. Thus, the XY DSD-A group and CAH group often undergo feminizing or masculinizing surgery in childhood and/or adolescence. In XY DSD-nA, the external genitalia phenotype is typically female.

**Table 1:** Sample description

	CAH		XY DSD, with androgen effect (A)		XY DSD, no androgen effect (nA)	
	*n* = 192		*n* = 150		*n* = 117	
	Salt-wasting (*n* = 111), simple virilising (*n* = 66), other forms of CAH (*n* = 15)^a^, M age (SD) 30.5 (10.9)		Partial GD (*n* = 37), ovotestes (*n* = 5) pAIS (*n* = 35), hypospadia (*n* = 25), androgen synthesis defects (*n* = 19)^b^, mixed GD (*n* = 21), other unknown XY DSD (*n* = 7), M age (SD) 26.0 (10.1)		Mixed GD (*n* = 24), complete GD (*n* = 21), cAIS (*n* = 71), other XY DSD (*n* = 1), M age (SD) 32,4 (13.8)	
	*n*	%	*n*	%	*n*	%
Gender						
Female	188	97.9	59	39.3	114	97.4
Male	4	2.1	87	58.0	0	–
Other^c^	0	–	4	2.7	3	2.6
Country						
Germany	74	38.5	36	24.0	29	24.8
France	58	30.2	36	24.0	24	20.5
The Netherlands	17	8.9	12	8.0	38	32.5
Poland	14	7.3	50	33.3	14	12.0
Sweden	12	6.3	13	8.7	10	8.6
United Kingdom	17	8.9	3	2.0	2	1.7
Genital surgery (missing *n* = 5)						
No	15	8.0	6	4.0	17	14.5
Yes	173	92.0	143	96.0	100	85.5
Clitoris surgery	129	68.6	36	24.2	1	0.9
Vaginoplasty	128	68.1	35	23.5	15	12.8
Hypospadias surgery	5	2.7	77	51.7	0	–
Contact to support group (last 12 months) (missing *n* = 24)						
No	163	88.6	120	87.0	89	78.8
Yes	21	11.4	18	13.0	24	21.2

*GD* gonadal dysgenesis, *pAIS* partial AIS, *cAIS* complete AIS

^a^Nonclassical CAH was not included in this study

^b^Androgen synthesis defects included 3β-Hydroxylase deficiency (*n* = 2) 17β-HSD III deficiency (*n* = 11), 5α-Reductase II deficiency (*n* = 4), 17α-Hydroxylase/17,20 lyase deficiency (*n* = 1), unknown androgen synthesis defects (*n* = 2)

^c^Inter (*n* = 4). open (*n* = 2). other (*n* = 1)

Data on DSD surgery (vaginoplasty, clitoris reduction, hypospadias repair) were collected by combining clinical report form and patient-reported data. Discrepancies between self-reported data and clinical report file were reviewed manually.

We classified gender as male, female, or other than male or female based on how participants identified at the medical interview.

### Measures

For the patient-reported outcome questionnaire, we developed items addressing the ethical evaluation of the need for and timing of genital surgery. We identified ethically contested issues via analysis of the relevant ethical and legal literature and via an explorative questionnaire sent to DSD support groups in associated countries as well as personal interviews with members of support groups. From this material, we constructed one general question and related answers and five statements to be rated on a five-item Likert scale. Three statements (3–5) referred to specific surgical procedures (clitoris reduction, vaginoplasty, hypospadia repair) relevant only for a part of the sample. To simplify the display of data on statements 2–6, we adapted answers from the 5-point rating scale (strongly/partly agree, neither agree nor disagree, partly/strongly disagree) into a 3-point one (agree, neither agree nor disagree, disagree).

1. “When do you think is the appropriate time for genital surgery?”
2. “Any decision about these surgical procedures should be postponed until the affected person reaches the age of legal responsibility.”
3. “Reduction of an enlarged clitoris is necessary in girls.”
4. “Vaginoplasty (surgical construction of a vagina) in adolescence or adulthood with patient’s consent is better than before 6 months of age (infancy).”
5. “Hypospadias repair with patient’s consent is better than before 2 years of age (infancy).”
6. “I think I would have been better off without any of the surgeries performed in my childhood/adolescence.”

### Data Analysis

In general, participants responses are displayed as percentages per diagnostic group, participant’s contact with support group within the last 12 months, gender, and/or history of genital surgery. For the XY DSD-A group, the association between responses and female or male gender was analyzed; results regarding persons with other gender are reported separately due to the small number of individuals concerned (*n* = 4); no association was analyzed between gender and CAH or XY DSD-nA due to the small number of individuals with male gender. All group differences were tested using Pearson *χ*^2^ tests*.* Due to the exploratory character of the analyses, all *p* values were interpreted as non-confirmatory and post hoc tests were omitted. SPSS Version 22 was used for all analyses.

## Results

### Sample

Medical diagnoses, age, gender, country, history of genital surgery, and contact with support groups are shown in Table 1. Response rate in the dsd-LIFE study was 36.1% (Rohle et al., 2017).

### General Postponement of Surgical Procedures until the Age of Legal Responsibility

**Fig. 1:**
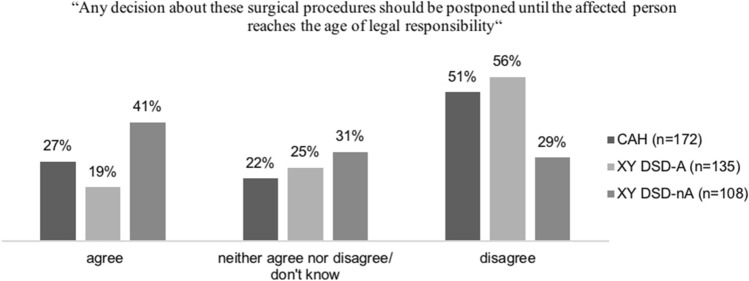
Participants’ views on postponing genital surgery until the age of legal responsibility

In total, 415 participants rated their agreement or disagreement with the statement “Any decision about these surgical procedures should be postponed until the affected person reaches the age of legal responsibility” (Fig. 1). In the CAH group, 88 (51.2%) participants disagreed with this statement; in the XY DSD-A group, 76 (56.3%) participants and 31 (28.7%) participants disagreed in the XY DSD-nA group. There was an association between views of participants and diagnostic grouping (*p* = .013). Participants with CAH or XY DSD-A were more inclined to disagree, whereas those with XY DSD-nA tended to agree with the statement.

### Appropriate Time for Genital Surgery

**Fig. 2:**
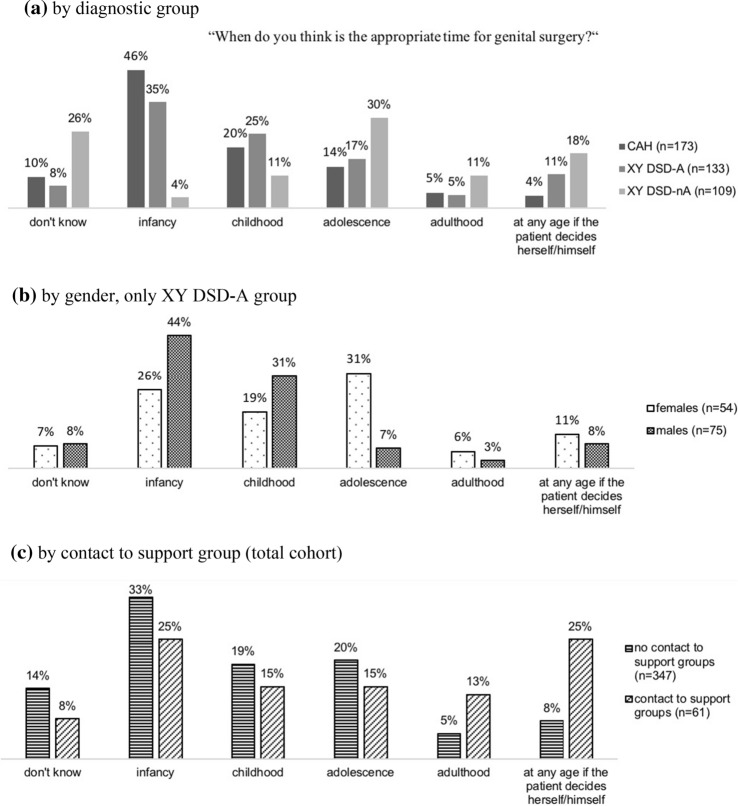
Participants’ views on the appropriate time for genital surgery. **a** By diagnostic group, **b** by gender, only XY DSD-A group, **c** by contact to support group (total cohort)

A total of 415 participants answered the question “When do you think is the appropriate time for genital surgery?” Altogether, 27 (6.5%) participants stated that adulthood was the right age for such a procedure. There was an association between participants’ choice of age and diagnostic grouping (*p* < .001) (Fig. 2a). Of the CAH group, 80 (46.2%) participants stated that infancy (1 month–3 years) was the appropriate age for genital surgery and 35 (20%) chose childhood (4–12 years). Of the XY DSD-A group, 47 (35.3%) participants preferred infancy and 12 (25%) preferred childhood. In contrast, participants in the XY DSD-nA group preferred adolescence (13–17 years; *n* = 33, 30%), adulthood (≥ 18 years; *n* = 12, 11%), or any age if the patient decides him-/herself (*n* = 20, 18%). Within the XY DSD-A group, there was an association between responses and gender (*p* = .007; Fig. 2b), since 44% of males preferred infancy and 31% childhood over other stages of life, whereas females slightly preferred adolescence (31%) over infancy (26%) and childhood (19%). There was an association between having had support group contact within the last 12 months and a preference for surgery at a later age (*p* = .001) (Fig. 2c).

### Clitoris Reduction

**Fig. 3:**
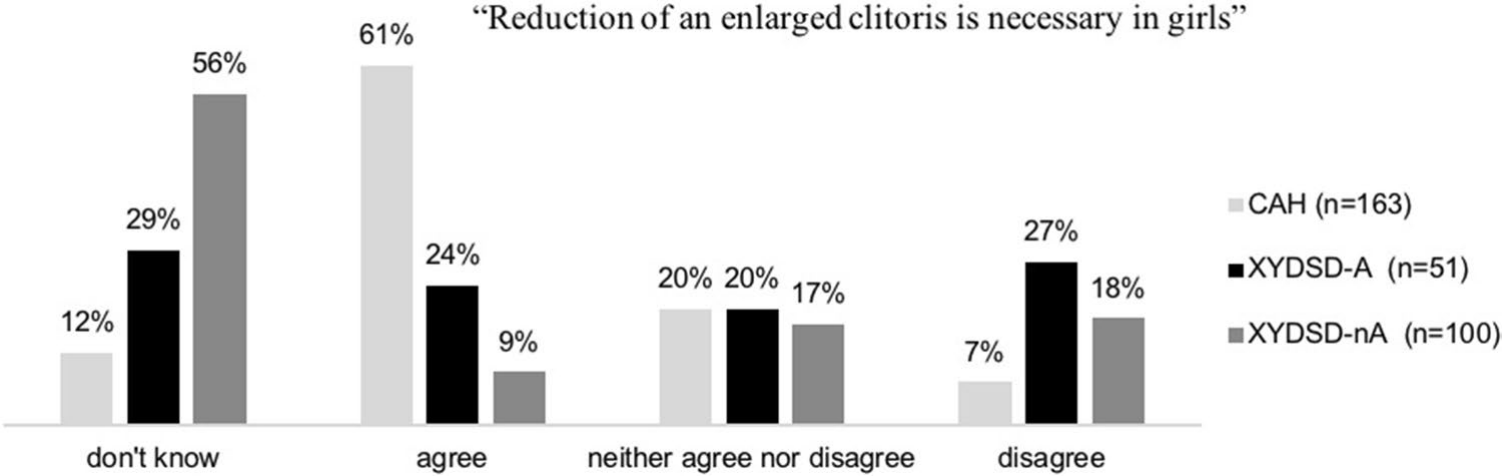
Views of participants with female gender on the need for clitoris reduction surgery

A total of 314 participants rated the statement “Reduction of an enlarged clitoris is necessary in girls” (Fig. 3, only persons identifying as female). Of those, 120 (38.2%) agreed, and 44 (14%) disagreed with this statement, 59 (18.8%) were undecided, and 91 (29%) answered “don’t know.”

There was an association between participants’ views and diagnostic grouping (*p* < .001) (Fig. 3). Most participants in favor of clitoris reduction were in the CAH group, whereas those undecided mostly belonged to the XY DSD-nA group. Those members of the CAH group who had had clitoris surgery more often preferred clitoris reduction in girls (*p* = .048) (see Table 2).

**Table 2:** Views of participants with female gender with and without a history of clitoris surgery on the need for clitoris reduction

	Females with CAH		Females with XY DSD-A	
	Clitoris surgery	No clitoris surgery	Clitoris surgery	No clitoris surgery
	*n* = 113	*n* = 30	*n* = 27	*n* = 21
	*n* (%)	*n* (%)	*n* (%)	*n* (%)
“Reduction of an enlarged clitoris is necessary in girls”				
Don't know	12 (10.6)	8 (26.7)	9 (33.3)	5 (23.8)
Agree	76 (67.3)	12 (40.0)	9 (33.3)	2 (9.5)
Neither agree nor disagree	20 (17.7)	6 (20.0)	6 (22.2)	4 (19.1)
Disagree	5 (4.4)	4 (13.3)	3 (11.1)	10 (47.6)

### Vaginoplasty

**Fig. 4:**
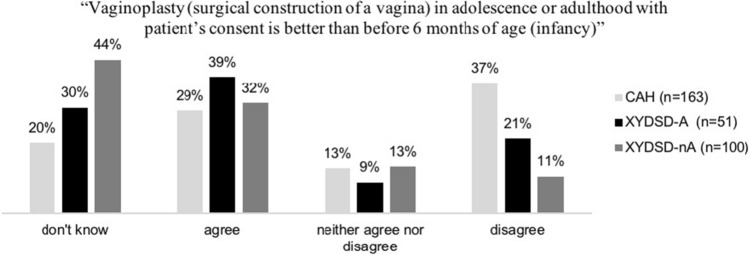
Views of participants with female gender on appropriate time of vaginoplasty

A total of 323 participants rated the statement “Vaginoplasty (surgical construction of a vagina) in adolescence or adulthood with patient’s consent is better than before six months of age (infancy)” (Fig. 4, only persons identifying as female). One hundred and three (31.9%) agreed, 40 (12.4%) neither agreed nor disagreed, and 84 (26.0%) disagreed with the statement (“don’t know”: 96/29.7%). There was an association between participants’ views and diagnostic grouping (*p* < .001), with participants in the CAH group tending to disagree with the statement (61/37%) more often than participants of the XY DSD-A and nA groups (12/21% and 11/11%). There was an association between participants’ views and history of surgery in the XY DSD-nA group (*p* = .014) (see Table 3).

**Table 3:** Views of participants with female gender with and without a history of vaginoplasty on the appropriate time

	Females with CAH		Females with XY DSD-A		Females with XY DSD-nA	
	Vaginoplasty	No vaginoplasty	Vaginoplasty	No vaginoplasty	Vaginoplasty	No vaginoplasty
	*n* = 113	*n* = 21	*n* = 30	*n* = 22	*n* = 14	*n* = 70
	*n* (%)	*n* (%)	*n* (%)	*n* (%)	*n* (%)	*n* (%)
“Vaginoplasty (surgical construction of a vagina) in adolescence or adulthood with patient’s consent is better than before 6 months of age (infancy)”						
Don't know	17 (15.0)	8 (38.1)	8 (26.7)	7 (31.8)	1 (7.1)	38 (54.3)
Agree	30 (26.6)	7 (33.3)	13 (43.3)	9 (40.9)	7 (50.0)	19 (27.1)
Neither agree nor disagree	13 (11.5)	2 (9.5)	3 (10.0)	2 (9.1)	4 (28.6)	5 (7.1)
Disagree	53 (46.9)	4 (19.1)	6 (20.0)	4 (18.2)	2 (14.3)	8 (11.4)

### Hypospadias Repair

**Fig. 5:**
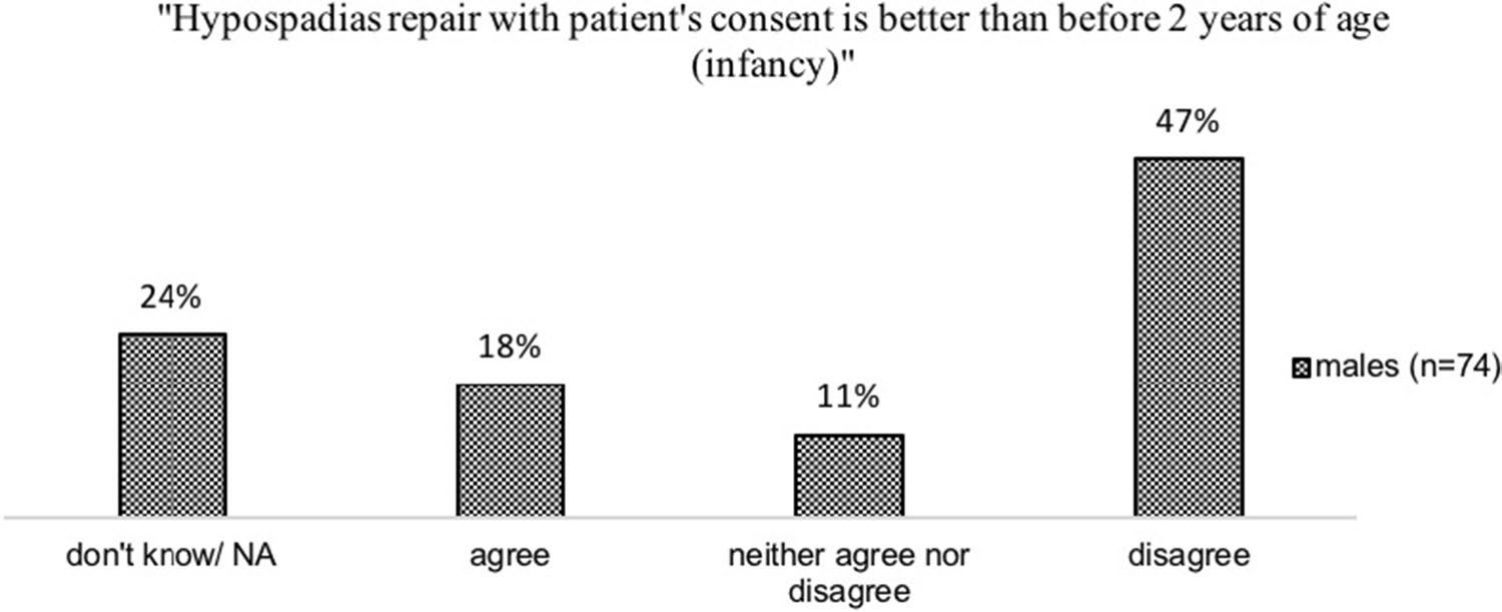
Views of participants with male gender on the appropriate time of hypospadias repair

Altogether, 74 participants who identified as male and had hypospadias surgery responded to the statement “Hypospadias repair with patient’s consent is better than before two years of age (infancy)” (Fig. 5, data are descriptive). A total of 13 (17.6%) participants agreed with the statement, eight (10.9%) neither agreed nor disagreed, and 35 (47.3%) disagreed.

### Views on Surgery, in General

**Fig. 6:**
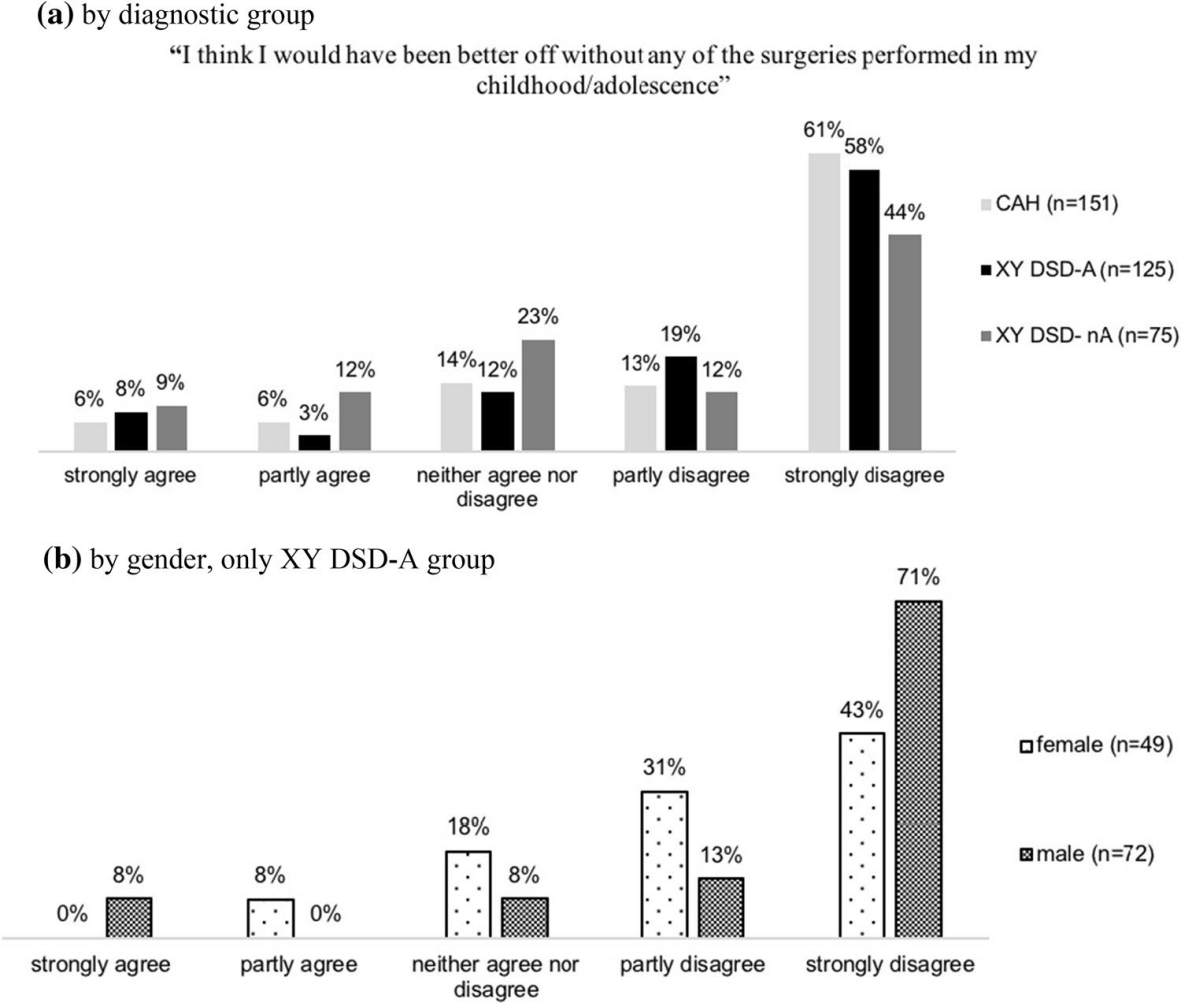
Participants’ views on surgeries performed in their childhood/adolescence. **a** By diagnostic group; **b** by gender, only XY DSD-A group

In total, 351 participants rated their level of agreement or disagreement with the statement: “I think I would have been better off without any of the surgeries performed in my childhood/adolescence.” A total of 48 (13.7%) participants partly or strongly agreed, while 250 (71.2%) partly or strongly disagreed with the statement, most of them strongly (197, 56.1%) (Fig. 6a). There was an association between views and diagnostic grouping (*p* = .001) since participants of the XY DSD-nA group were less inclined to disagree with this statement.

## Discussion

### Principal Findings

Our data show that persons with DSD have a much more differentiated view on early genital surgery than might be deduced from current publicized views. The evaluation of the appropriate time for the surgery of ambiguous genitalia differed significantly between the groups with CAH, XY DSD with and without androgen effect (A/nA). A total of 66% of individuals with CAH and 60% of individuals with XY DSD-A said that infancy or childhood was the appropriate age for genital surgery, whereas 23% and 33%, respectively, preferred adolescence or adulthood, or any time if the patient decides herself/himself. Individuals in the XY DSD-A group who identified as males were more likely (75%) than persons living in the female gender (45%) to favor infancy or childhood. In general, individuals who had early genital surgery were more likely to approve of it than those who had not. Only 12% of participants from the CAH group, 11% of the XY DSD-A group, and 21% of the XY DSD-nA group said that they would have been better off without any surgeries performed in their childhood or adolescence, thus demonstrating the prevalent acceptance of surgical interventions, independent of timing.

Participants with 46,XX CAH living in the female gender (a population that usually has vaginoplasty and/or clitoris reduction early in life) predominantly (66%) approved of surgery in infancy or childhood. Slightly more individuals thought that surgical construction of a vagina was better done in infancy than in adolescence or adulthood with patient’s consent (37% vs. 29%). Nowadays, some clinicians tend to postpone the time of vaginoplasty to adolescence because it is considered functional only during or after puberty; others recommend infancy, based on anatomical patterns that seem to promise better outcome (Creighton, 2005; Creighton, Chernausek, Romao, Ransley, & Salle, 2012). Our results indicate that surgeons should continue to seek functional outcome data as well as explore their patients’ rationale which may differ from their own evaluation.

The majority of individuals with CAH (61%) and 24% of individuals of the XY DSD-A group agreed that a reduction of an enlarged clitoris was necessary in girls, an operation that is highly contested in the DSD debate, since this type of surgery is understood as having mainly cosmetic function and carries the risk for complications like reduced sensitivity. Those who had clitoris surgery themselves were more likely to approve of clitoris reduction in girls. More qualitative research is needed to understand personal motives; one reason may be that from a subjective point of view, for females with CAH, typical female appearance might signify more than just cosmetic improvement, as it may be more closely associated with bodily integrity and appropriate functioning. Moreover, the views of this particular group were probably not adequately represented in the public discourse because a large number of adult persons with CAH do not identify as intersex or having a DSD later in life, although they are often born with ambiguous genitalia and early genital surgery is performed in this group. For example, in an online survey by the German Ethics Council from 2011, nearly all participants with CAH responded that they did not feel that they were intersex (German Ethics Council, 2012). As a consequence, their preferences are less likely to be taken into account.

Persons with male gender and XY DSD who had hypospadias surgery also predominantly agree that hypospadias repair is better done in infancy (47.3%). This may be the case because severe instances of hypospadias in boys can it make difficult to urinate upright and cause significant shame and humiliation (Rynja, de Jong, Bosch, & de Kort, 2011). But many (35%) were undecided, while 18% preferred a later age. More qualitative research is needed to explore the underlying motives of the persons concerned.

Many persons in the XY DSD groups were undecided on the issue of interventions in infancy or childhood. It is noteworthy that, although some advocacy groups have tirelessly campaigned for a general moratorium, the persons in our study still mostly have not made up their minds. Overall, individuals tend to favor the age at which they underwent the surgery they experienced in their own lives.

Yet, it should be made clear that the justification of elective genital surgery in early childhood is fundamentally an ethical problem; solutions for an ethical problem should not simply be based on the attitude of majorities (Mertz et al., 2014; Salloch, Vollmann, & Schildmann, 2014). Obviously, the large variety of views expressed within each DSD group indicates a fundamental conflict of values that must be addressed. Notwithstanding these cautionary remarks, and precisely for the same reasons, a general moratorium would not be justified either. Patient autonomy is rightly claimed to play a major role in medical decision-making, and, unfortunately, very young children cannot exercise autonomous decision-making. However, at the very least, the diversity of options preferred by adult patients should be considered when a moratorium is discussed at the legislative level.

The framing of the underlying conflict by some political institutions and advocacy groups that favor a ban is problematic since it fails to acknowledge the complexity of the issues at stake. Critics maintain that elective surgery in DSD is “medically unnecessary” and must therefore be postponed until the age of informed consent. Yet, the bioethicist Morreim (2001) warns that medical choices, in general, mostly “require a level of clinical complexity that is not reflected in simplistic notions like necessity, and that should not be hidden under blanket categories connoting a façade of precision.” Even life-saving treatments for cancer are evaluative in the sense that different options have to be weighed against each other. This is precisely the realm where patient-reported outcome and patient preferences can make all the difference.

### Comparison with Other Studies

Other studies on patient evaluation of the appropriate timing of elective genital surgery, in general, support our findings. However, samples sizes were too small to allow for generalizable conclusions. Most refer to only one DSD condition, employ a strictly local approach, and/or often included just one question regarding the relevant ethical aspects. In a retrospective evaluation of 55 adult XY DSD patients (age range 18–60), 67% did not agree that corrective surgery should generally be postponed to adulthood (Meyer-Bahlburg et al., 2004). In a questionnaire-based survey from the Johns Hopkins School of Medicine on women affected by CAH due to 21-hydroxylase deficiency, patients were asked when feminizing surgery should be performed (age range at participation, 21 to 71). Fifteen out of 31 chose infancy and nine toddler age or elementary age (Wisniewski, Migeon, Malouf, & Gearhart, 2004). A follow-up study on 62 women with CAH from Sweden found that 20 patients preferred early timing; however, 34 patients did not answer this question (Nordenskjold et al., 2008). In a survey from Finland, of 24 patients 19 had undergone clitoris reduction and 21 reconstruction of the vaginal introitus in childhood. None of them believed that the operation was performed too early (Fagerholm et al., 2011). In a French questionnaire-based survey with 21 patients with CAH (M age, 27) who all had had clitoris reduction and vaginal surgery 90% said that genitoplasty should be performed during the first year of life (Binet, Lardy, Geslin, Francois-Fiquet, & Poli-Merol, 2016).

### Strengths and Limitations

The study was part of a large European quantitative cross-sectional outcome study and, to date, the largest and most comprehensive study evaluating (former) patients’ views. Major ethical aspects were included in study design, recruitment of participants, and interpretation of data. In this study, it was not asked whether participants knew about the ethical debate on early genital surgery, so they might have been biased toward surgery as the only possible solution. Furthermore, a selection bias cannot be ruled out, as participants were partly contacted through medical personnel, a fact that might have suggested compliance with medical authority in these debates. Also, the title of the study “dsd-LIFE” may have deterred those who object to the term “disorders of sex development.” In DSD, loss to follow-up is frequent, so we may have predominantly attracted the “care-minded” and possibly more satisfied patients.

### Conclusions and Policy Implications

Our data suggest that a general moratorium on elective genital surgery in early childhood would not appropriately reflect the views of persons with DSD. This is notably true for persons with CAH and for persons with XY DSD and hypospadias who predominantly favor interventions in childhood. Persons with XY DSD living in the female gender are divided on this issue with many having no clear-cut view. Thus, our data from patient-reported outcome do not justify a straightforward, one-for-all solution of the ethical dilemmas involved in DSD medical treatment, as a general moratorium would entail. Case-by-case decision making, as suggested by several ethical boards (German Ethics Council, 2012; Gillam, Hewitt, & Warne, 2010; Karkazis, Tamar-Mattis, & Kon, 2010; Wiesemann, Ude-Koeller , Sinnecker, & Thyen 2010), is better suited to grasping the remarkable variety of (former) patients’ views, the very complexity of the issues at stake, and the ethical values underlying elective genital surgery in early childhood. Instead of promoting a polarizing moratorium, more efforts should be invested in improving information on long-term outcomes, informed consent and assent, and contact with support groups on both the individual and the institutional level to support decision making. Overall, the considerable uncertainty about the best time for genital surgery, if considered at all, among (former) patients justifies, if not a ban, then at least deliberate attempts to motivate parents to postpone elective genital surgery, thus relieving themselves as much as possible of the responsibility for taking the “right” choices.
